# Health professionals’ licensing: the practice and its predictors among health professional hiring bodies in Ethiopia

**DOI:** 10.1186/s12960-022-00757-6

**Published:** 2022-08-19

**Authors:** Endalkachew Tsedal Alemneh, Biruk Hailu Tesfaye, Eshetu Cherinet Teka, Firew Ayalew, Ermias Gebreyohannes Wolde, Wondimu Daniel Ashena, Tewodros Abebaw Melese, Fikadie Dagnew Biset, Bezawit Worku Degefu, Bethlehem Bizuayew Kebede, Yohannes Molla Asemu, Meron Yakob Gebreyes, Wudasie Teshome Shewatatek, Samuel Mengistu, Tangut Dagnew, Yeshiwork Eshetu Abebe, Matias Azanaw Aliyu, Fatuma Ahmed Endris, Eden Workineh Sahlemariam, Genet Kifle Weldesemayat

**Affiliations:** 1 Health Professionals’ Competency Assessment and Licensing Directorate, Ministry of Health Ethiopia, Addis Ababa, Ethiopia; 2 Johns Hopkins Program for International Education in Gynecology and Obstetrics, Addis Ababa, Ethiopia; 3 Ethiopian Medical Association, Addis Ababa, Ethiopia

**Keywords:** Licensing, License, License practice, Health professionals, Hiring bodies, Human resources

## Abstract

**Background:**

Evidence suggests that not all human resource departments have hired their facility staff based on federal licensing standards, with some hiring without an active license. This is common in some, if not all, parts of the country. The paucity of healthcare experts, high turnover rates, employee burnout, and challenges in training and development issues were all key recruiting challenges globally.

**Objective:**

To assess the practice of health professionals’ licensing and its predictors among hiring bodies in Ethiopia, March 24/2021–May 23/2021.

**Methods:**

A cross-sectional study was conducted in privately and publicly funded health facilities throughout Ethiopia. For each region, a stratified sampling strategy was utilized, followed by a simple random sampling method. Documents from the recruiting bodies for health professionals were reviewed. A pretested structured questionnaire and document review tool were used to extract data confidentially. A descriptive analysis of the basic hiring body characteristics was conducted. Hiring body characteristics were analyzed in bivariate and multivariate logistic regression to identify factors associated with best health professionals licensing practice. Data management and analysis were conducted with Epi-Data version 4.4.3.1 and SPSS version 23, respectively.

**Results:**

The analysis included 365 hiring bodies and 4991 files of health professionals (1581 from private and 3410 from public health organizations). Out of 365 hiring bodies studied, 66.3% practiced health professional licensing. A total of 1645 (33%) of the 4991 professionals whose files were reviewed were found to be working without any professional license at all. Furthermore, about 2733 (55%) have an active professional license, and about 603 (12%) were found to work with an expired license. Being a private facility (adjustedOR = 21.6; 95% CI = 8.85–52.55), obtaining supervision from a higher organ (adjustedOR = 19.7; 95%CI: 2.3–169.1), and conducting an internal audit (adjustedOR = 2.7; 95% CI: 1.15–6.34) were predictors of good licensing practice.

**Conclusions:**

The licensing of health practitioners was poorly practiced in Ethiopia as compared to the expected proclamation of the country. A system for detecting fake licenses and controlling revoked licenses does not exist in all regions of the country.

## Background

The World Health Organization (WHO) under its “Global Strategy on Human Resources for Health 2030” acknowledges the following as one of its objectives: “To optimize performance, quality and impact of the health workforce through evidence-informed policies on human resources for health” [1].

As we inhabit the current fast-paced period, we are confronted with new infectious, environmental, and behavioral hazards, as well as rapid demographic and epidemiological transitions that jeopardize everyone's health and security. With these issues, it's clear that the healthcare system is failing to keep up, becoming more complex and expensive, and putting more strain on healthcare workers [2].

To make the matter worse, professional education does not appear to be keeping up with these problems, owing to fragmented, obsolete, and static curricula that generate ill-prepared graduates [3]. As a result, unless and until an adequate and proper regulatory structure is established and implemented, the first steps toward assuring a well-resourced healthcare system will not be evident anytime soon [3].

Putting in place a regulation can be a powerful weapon and policy instrument for safeguarding the public from unqualified, inept, or dangerous health care professionals [4].

While most countries have their scope and organizational arrangement when it comes to setting up health professional regulations, it is common for all to start by maintaining a register or list of those who are registered, setting educational standards as well as the scope of practice, establishing an ethical review system, relicensing previously registered professionals, and ensuring continued competence to practice [5, 6].

Regardless of wide global variations, most countries and areas have legislation that regulates medical doctors, dentists, nurses, midwives, and, often, pharmacists; a few countries and areas separately regulate some allied health professions (e.g., China, Hong Kong (SAR), New Zealand and Singapore) [7–9]; and a small number of countries and areas regulate some traditional medicine professions (e.g., Australia, Japan, and the Republic of Korea) [10].

In Ethiopia, the government established the Food, Medicine, and Healthcare Administration and Control Authority (FMHACA) in 2010 with the mandate to protect population health by ensuring the competence and ethics of health professionals [11]. Since then, the FMHACA and its branches have been managing health professional registration and licensing, the scope of practice, ethics, and continuing professional development [11]. Furthermore, Health professionals’ licensing was governed by the FMHACA proclamation (661/2009) up to the end of 2018 and this mandate is disaggregated into food and drug administration, clinical service regulation, and health professional regulations (Proclamation No.1112/2019).

The Health Professionals' Licensing Examination was put in place in July 2019 by the Ministry of Health Ethiopia being considered a critical step that should be undertaken for licensing new graduates [12].

It is stated that the major challenges faced at the Human Resource (HR) level hindering proper recruitment and hiring are mostly attributed to the scarcity of healthcare professionals, a high attrition rate of professionals, employee burnout, and challenges in training and development [11, 13].

It is illegal to practice without a license in Ethiopia and most other nations [9, 11]. Individuals who provide medical, nursing, or other professional services without the required qualification or license may face penalties in most jurisdictions, including criminal charges and imprisonment [9, 11].

Although Ethiopia adopted various health professional regulatory frameworks, their implementation has fallen behind due to several factors one of them being the gap visible in the step-down application of hiring only licensed professionals at the health-care facilities.

To the best of our knowledge, little or no evidence exists in Ethiopia about the practice of hiring bodies of licensed health professionals. Furthermore, no evidence of the causes that led to the hiring of these unlicensed professionals has been found. This context necessitates primary research.

Therefore, this study is primarily aimed to determine the proportion of hiring bodies with good licensing practices in Ethiopia from March 24/2021 to May 23/2021. Moreover, the study aimed to assess the knowledge and attitude of hiring body managers on health professionals licensing practice in Ethiopia and identify the predictors of health professionals' licensing practice among hiring bodies in Ethiopia from March 2021 to May 2021.

## Methods

### Design and period

A cross-sectional study design was utilized with a structured questionnaire, and files from each selected health institution, district health office, zonal health department, and regional health bureau were reviewed. The study was carried out between March 24th and May 23rd, 2021.

### Area and setting

This study was conducted at selected health institutions, district health offices, zonal health departments, and regional health bureaus in all regions of Ethiopia.

By January 2021, In Ethiopia, there were 381 public hospitals (including specialty, referral, general and primary hospitals), 3362 health centers, 23 private hospitals (primary hospitals & general hospitals), 587 specialties (specialty centers and specialty clinics) 3274 private medium and lower clinics, 12 regional health bureaus (including city administrations), 75 zonal (special woreda) health departments, and 700 district health offices [14]. Each health facility and health sector have its human resource manager responsible for the selection and recruitment of health professionals.

### Population

The study units were randomly selected health professional hiring bodies in Ethiopia. Moreover, a document review of health professionals hired at specialized, general, and referral hospitals was done by only considering recruitments after the implementation of the licensure examination in the country (Since July 2019). Furthermore, all files, despite the year of recruitment, of health professionals working in other sorts (Health centers, private facilities, etc.) of health institutions were reviewed.

### Inclusion and exclusion criteria

#### Inclusion criteria

-  Human resource managers who were working in health facilities, district health offices, zonal health departments, and regional health bureaus were included in the study.
-  Health institutions that were given health care services with the mandate to recruit health professionals were included in the study.

#### Exclusion criteria

- Institutions that had duplicated files of professionals found in higher level recruiters, such as regional health bureaus.
- Diagnostic centers and pharmaceutical services providers were excluded from the study
- Files of health professionals recruited before July 2019 at specialized, referral, and general hospitals were excluded from the study.

## Sampling and sample size

### Document review sampling

All files of health professionals hired after June 2019 were reviewed at government-owned specialized hospitals, referral hospitals, and general hospitals. A random sample of 30 files was also taken for regional health bureaus and zonal health departments. All files of health professionals were considered in the case of files from general hospitals and health facilities.

### Hiring bodies sampling

The sample size for the quantitative interview was calculated using a single population proportion formula based on the following assumptions. For an average effect size of 50% with a 5% margin of error and a normal assumption of the distribution around the effect size [15, 16], a sample size of 384 was required to use the hiring bodies' units of analysis.

A stratified sampling technique by region followed by a simple random sampling technique (lottery method) was applied to select the hiring bodies. Sample for hiring bodies in each region was allocated proportionally. One participant (human resource manager) was selected from each hiring body.

## Data collection methods

This study underwent a data collection process using two types of tools, namely, a document review checklist and a structured interview questionnaire.

### Document review checklist

Document review was the first step in the data collection process. After a thorough explanation of the objective of the study, data collectors visited the hiring body’s record and archive room before distributing the quantitative questionnaire.

### Quantitative interview questionnaire

Quantitative data were collected using a structured questionnaire filled out by data collectors. The questionnaire consisted of close-ended questions containing basic characteristics of respondents, basic characteristics of hiring bodies, knowledge, attitude, and practice-related questions on licensing of health professionals. After explaining the purpose of the study and obtaining written informed consent, data collectors interviewed and filled out the structured questionnaire accordingly.

### Outcomes of the study

Licensing practice of health professionals among hiring bodies in Ethiopia is the primary outcome of this study, which is ascertained by the proportion of hiring bodies that have complete (100%) documents of their professionals with an active license.

The proportion of health professionals with an active license in the study period and predictors of health professionals' licensing practice among hiring bodies are the secondary and tertiary outcomes of interest in this study.

### Data quality assurance

Before deployment, data collectors and supervisors received training in performing structured interviews and document reviews. Field supervisors rechecked completed questionnaires for clarity and completeness immediately after collecting data at the field level and upon submission to the HPCALD to ensure data quality.

The questionnaire was translated into a local language (Amharic) during data collection and then back to English for data entry. To keep the tool consistent, a language specialist back-translated the questionnaire into English. Both tools were pretested with five percent of study participants, hiring bodies who worked at Debre Birhan town, who were thereafter excluded from the study. The data-gathering instruments were then modified and reconfigured to collect the study's desired data. Data were collected by lecturers from higher education institutions, and the process was overseen by 20 HPCALD employees (health professionals).

### Data analysis

Data were entered into Epidata version 4.4.3.1 [17] and were cleaned and analyzed by SPSS version 23 [18]. Descriptive statistics were reported using frequencies, proportions, cross-tabulations, and mean for the basic characteristics of the participants and then presented using tables and graphs. A logistic regression analysis was also conducted, and associations with a significance level of less than 0.05 were considered for multivariate regression analysis. Odds ratios (OR) and adjusted OR with 95% confidence intervals (CI) were used to determine the strength of associations between the licensing practice and its predictors by taking the health professionals' documents as a unit of analysis. Moreover, a stratified analysis was done by region and profession category among the hiring bodies to identify the status of the license of health professionals.

### Definition of terms

- Hiring body/recruiter: an entity authorized or responsible to recruit and deploy health professionals at regional, zonal, district and health facilities of Ethiopia.
- Good licensing practice: a practice where all health professionals in the respective hiring body had a license attached to their files for 100% of the expected professional categories.
- Poor licensing practice: a practice where < 100% of health professionals in the respective hiring body had a license attached to their files.
- License: a document given by a regulatory body that permits a particular health professional to practice [19].
- Health facility/Institution: hospitals, health centers, clinics; excluding health posts, diagnostic centers, pharmacies, and higher education institutions (health).
- Human Resource Managers: A person responsible to recruit and deploy health professionals.
- Regulatory Body: An organization responsible for registering and licensing health professionals in Ethiopia [19].
-  Certificate of Competency (COC): a certificate given for a diploma graduate by the TVET agency in Ethiopia.
- Licensure Examination: is a competency assessment given for bachelor-level health professionals as a prerequisite to being licensed in Ethiopia [12].

## Results

### Findings from health professional hiring bodies

#### Socio-demographic characteristics of study participants

A total of 365 human resource managers took part in the study, yielding a 95% response rate. Females made up three out of four respondents (78.9%). The respondents' mean age was 40.67 years with a standard deviation of 10.1 (95% CI: 39.58, 41.74) (Table 1).

**Table 1:** Sociodemographic characteristics of the participants of health professional hiring bodies of Ethiopia, 2021

Characteristics	Number (*N* = 365)	%
*Age of respondents (in years)*		
21–30	44	12.1
31–40	141	38.6
41–50	103	28.2
> 50	77	21.1
*Educational status*		
Second degree and above	69	19.2
First Degree	248	67.7
Diploma	42	11.5
Secondary education and below	6	1.6
*Job responsibility*		
Director/Deputy	144	39.5
Team coordinator	137	37.5
Professional	29	16.2
Other	25	6.8
*Decision-making role in health professionals' hiring*		
Very High	174	47.6
High	154	42.2
Low	14	3.8
Very low	5	1.4
No role	18	4.9
*Total work experience (in years)*		
< 5	19	5.2
6–10	60	16.4
11–15	96	26.3
16–20	63	17.3
21–25	39	10.7
> 25	88	24.1
*Work experience in this position (in years)*		
< 5	179	49.0
6–10	93	25.5
11–15	44	12.1
16–20	10	2.7
21–25	6	1.6
> 25	33	9.0

#### Basic characteristics of hiring bodies

**Fig. 1:**
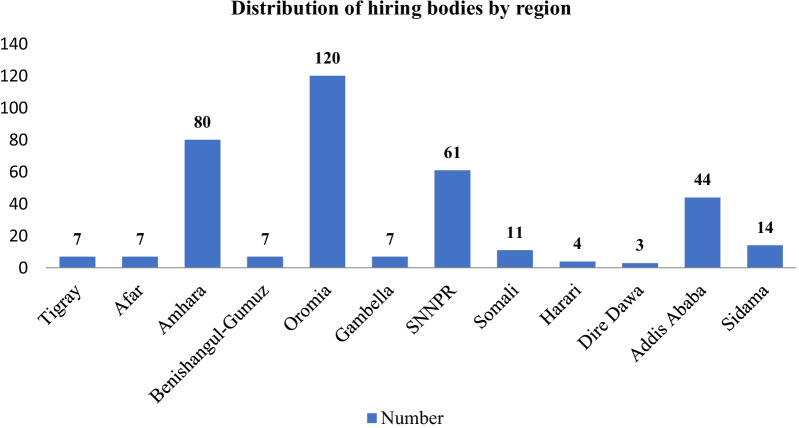
Frequency distribution by region of the studied health professional hiring bodies of Ethiopia, 2021

Most health professional hiring bodies (56.2%) were privately held, with the rest being governmental institutions (Fig. 1).

**Fig. 2:**
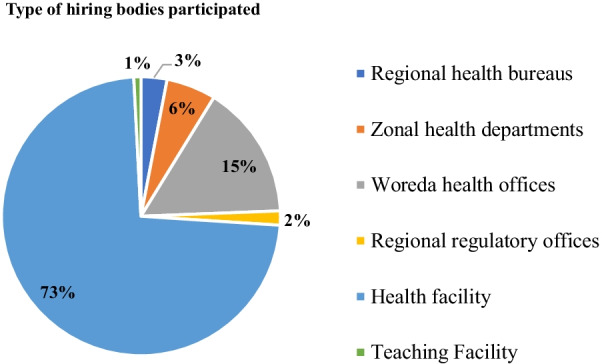
Type of health professional hiring bodies included in the study, Ethiopia, 2021

The majority (73%) of the health professional hiring bodies that took part in this study were from health facilities of various levels and ownership types (Fig. 2).

#### Health professionals’ hiring structures and readiness

Two hundred and eighty-eight (78.9%) of the respondents believed that they had a well-organized health professional hiring system, while 61 (16.7%) said they do not have a well-structured hiring system, and 16 (4.4%) said they do not have a structured health professional recruitment system at all in their institution. The hiring of health professionals was confirmed by the director in the majority of hiring bodies (63.5%), the human resource head (24.1%), and the team leader (12.1%).

### Internal human resource audit

**Fig. 3:**
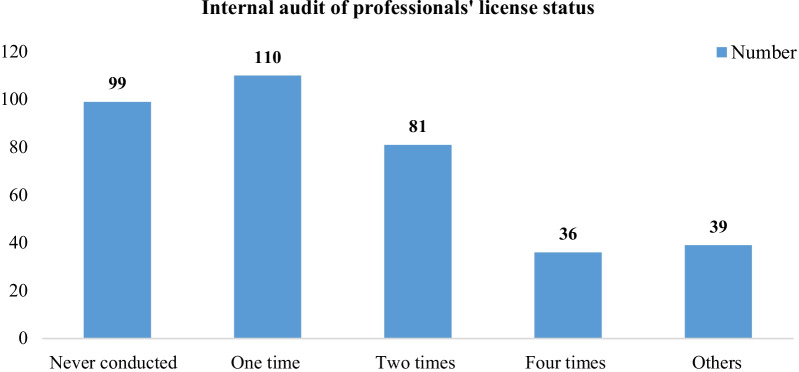
Frequency of internal human resource audit on professionals’ license status among hiring bodies of Ethiopia, 2021

Internal human resource audit was done by 110 (30.2%) of the facilities at least once a year and reviewing the records was the commonest method or mechanism of audit reported by 227 (62.2%) of the participants in the institution (Fig. 3).

### Knowledge and attitude on health professionals’ licensing

Three hundred and fifty-eight (98.0%) of the respondents said that they have heard or read about health professionals’ licensing in the country. In addition, 120 (33.41%) of those who have ever heard about licensing, regulatory bodies were the main source of information.

Most human resource managers (88.2%) said that they had not received any training about health professionals' licensing. A professional license was a priority requirement for 327 (89.5%) of the hiring bodies when it came to hiring health professionals. Most of the respondents (77%) said they had the information on health professionals' licenses being revoked. Furthermore, the majority of participants, 312 (85.5%), thought that the licensure of health professionals was very important (Table 2).

**Table 2:** Perception of health professional hiring bodies on the benefit of health professionals’ licensing of Ethiopia, 2021

	Number	%
*The benefit of health professionals' license*		
Very important	312	85.5
Important	50	13.7
No benefit	1	0.3
*Contribution of being licensed to the quality of health care*		
Have contribution	361	98.9
No contribution	4	1.1

### Health professionals' licensing practice among hiring bodies

One-fifth of recruiting bodies (20.55%) had experience in hiring health professionals without a license. Moreover, 136 (37.3%) of the health professional hiring bodies had a mechanism in place to verify the license's originality, of which, 40 (29.4%) said they found fake licenses during recruitment. Fifty-seven (15.62%) of respondents had seen a health professional being recruited without a license in their institution, and 40 (10.9%) had taken part in the recruitment of health professionals without a license. Two hundred and eleven (60.5%) of the hiring bodies had never attended or conducted training about health professionals' licenses in their institution.

**Fig. 4:**
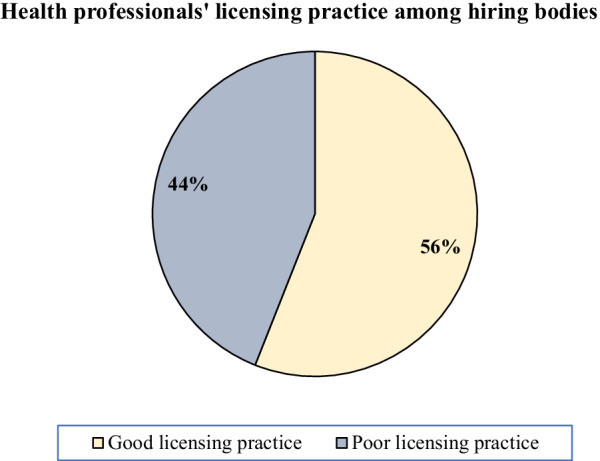
Health professionals licensing practice among hiring bodies of Ethiopia, 2021

About 56% of the health professional hiring bodies of Ethiopia were found to have good licensing practices and the rest 44% have poor licensing practices while recruiting health professionals in their institutions (Fig. 4).

### Findings from health professional documents review

#### Licensing status of health professionals

**Fig. 5:**
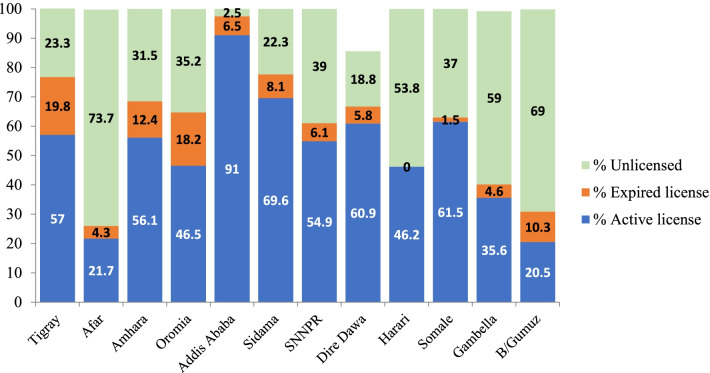
Licensing status of health professionals by region among hiring bodies of Ethiopia, 2021

The files of 3338 (66%) of the 4991 health professionals reviewed have active licenses at the national level. However, the findings vary between regions. In the Benishangul–Gumuz region, only 20% of health professionals had an active license and Addis Ababa had 91% of professionals with an active license (Fig. 5).

#### Status of health professionals’ license by profession

**Fig. 6:**
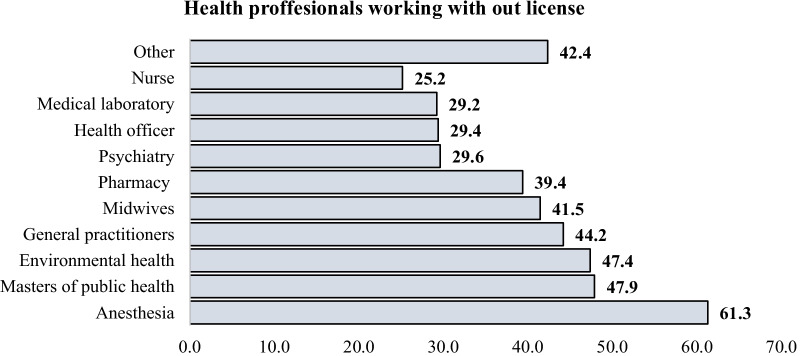
Proportion of professionals’ working without a license by profession among hiring bodies of Ethiopia, 2021

In various parts of the country, over 61% of Anesthesia practitioners practice without a license or with an expired license. On the contrary, from the reviewed documents of professionals, only about 25% of Nursing practitioners work without a license (Fig. 6).

#### Determinants of licensing practice in Ethiopia

Among variables that showed association on bivariate logistic regression, organizational ownership, being supervised by higher organs, and conducting internal audits showed significant association with health professionals’ licensing practice in binary multivariable logistic regression.

Private health facilities were 21 times more likely to have good licensing practices than public health facilities (adjusted OR = 21.6; 95% CI = 8.85–52.55) (Table 3).

**Table 3:** Determinants of health professionals' licensing practice among health professional hiring bodies of Ethiopia, May 2021

Variables		Licensing practice		Crude OR (95% CI)	Adjusted OR (95% CI)	*P* value
		Good	Poor			
Ownership	Private	93	112	11.23 (5.75, 22.00)	21.6 (8.85, 52.55)	0.001
	Public ^a^	11	149	1.00		
Supervision and technical support from higher organ	Yes	85	179	2.37 (1.18, 4.77)	19.7 (2.3, 169.1)	0.006
	Yes, but not continuous	11	55	1.6 (0.69, 3.68)	9.6 (0.2, 67.7)	0.03
	Not at all ^a^	8	27	1.00		
Conducting an internal licensing audit	Yes, four times per year	17	19	2.25 (1.1, 4.65)	2.7 (1.15, 6.34)	0.023
	Yes 1 to 2 times per year	53	133	2.8 (1.34, 6.13)	1.3 (0.75, 2.28)	0.339
	No internal audit ^a^	34	109	1.00		
Work experience of respondents	Less than 10 years	26	53	1.2 (1.67, 2.16)	2.7 (0.96, 7.42)	0.06
	10–20 years	46	113	1.46 (1.79, 2.67)	3.2 (0.41, 7.19)	0.005
	> 20 years ^a^	32	95	1.00		
Number of supervision and technical support from higher organ	3 to 5 per year	60	129	1.45 (1.88, 2.4)	10.2 (0.52, 68.0)	0.017
	1 to 2 per year	32	100	1.24 (1.59, 2.58)	14.9 (0.18, 101.54)	0.006
	No supervision at all ^a^	12	32	1.00		

^a^Reference category

When compared to hiring bodies that did not receive supervisory support from the responsible higher organs, hiring bodies that received supervisory support were 19 times more likely to have good licensing practice (adjusted OR = 19.7; 95% CI: 2.3–169.1) (Table 3).

Hiring bodies that perform professional internal audits four times a year had two times higher probability to have good licensing practice (adjusted OR = 2.7; 95% CI: 1.15–6.34) compared to those that did not perform an internal audit (Table 3).

## Discussion

The primary goal of this study was to assess health professional licensing practices among hiring bodies at various levels of Ethiopia's health care system. According to the Ethiopian health professional's regulation act, article 55 (3) proclamation no 661/2002 and revised article 73 of proclamation number 1112/2011, all health professionals working in the country's health system shall have a license and have it attached to their records [19, 20]. In contrast to this assertion, our research indicated that half of the health professional hiring bodies still have poor license practice, and licenses not being as an essential requirement for health professional employment. Furthermore, just a third of the country's professionals are registered with regulatory bodies, implying that there is a gap to be improved by all health professional regulatory bodies in Ethiopia.

Other key aspects of good licensing practice, such as awareness of expected professions to have a license, forgery control mechanism, and recognizing and regulating a revoked license [20, 21], were shown to be below the standards in our study findings. This is in accordance with the findings of another study conducted in Ethiopia [11]. This means that even while the hiring bodies are aware of and believe that a license is necessary for health professionals’ recruitment, their level of commitment falls short of what the regulations and laws require.

Another objective of this research was to find out what factors were linked to the licensing—practice of hiring bodies at various levels in Ethiopia. When compared to public-owned hiring bodies, private-owned hiring bodies were more likely to have good licensing practices, and our triangulation with the document review verifies this finding.

As a result, the ownership of the institutions might influence their licensing practices which could be due to strict enforcement of the regulation at these establishments. This theory is also supported by other similar evaluation assessments of private health facilities in Ethiopia [22, 23].

The implementation of any program in a country is expected to be monitored and evaluated in a variety of ways to ensure its long-term viability and quality improvement to meet the key objectives it set out to achieve [24, 25].

Our study elucidates that health professional hiring bodies that received timely supervision were more likely to have good licensing practices than those continuously unsupervised health professional hiring bodies. Our finding is in line with other studies conducted on the effectiveness of supportive supervision in other health system functions [26, 27]. This implies that having supervision from a higher authority has a strong link to effective licensing practice, highlighting the necessity for various responsible bodies to focus on strategies to sustain and strengthen the existing supervision from regional and federal health system regulatory.

Human resource audits are essential for avoiding legal and regulatory responsibility arising from an organization's human resource policies and initiatives, as well as achieving and maintaining world-class human resource practices [28]. Furthermore, one of the key goals of human resource auditing is to ensure the organization's compliance with federal rules and regulations [29]. Our finding showed that health professional hiring organizations that perform internal audits are three times more likely than those that do not have appropriate licensing practices. This means that the practice of human resource internal audit assists the organization in achieving and maintaining its human resource policies and strategies, which are mostly derived from regional and federal regulators. Moreover, the most important aspect of an internal audit is that it is self-initiated and requires minimal financial resources.

## Limitations of the study

Our research has two limitations. The first limitation is that the study design prevented us from observing how the licensing practice affected health professionals and healthcare institutions, hence we could only determine the primary outcome through document review. This study attempted to confirm the licensing practice with a document review of the professionals working in each facility rather than just the response of the hiring bodies despite the sensitive nature of the licensing practice, which is expected to be 100% applicable by the country's laws and proclamations. Therefore, a more robust causal-effect relation study is highly recommended to understand the effect on the broader health professional regulation stakeholders of the country. Another limitation of our study is that, despite exceeding the minimum sample size requirement, some institutions lacked professional documents, making it impossible to ascertain their exact position in regional analysis and reducing our sample size.

## Conclusions

In comparison to the expected full practice of licensing for health professionals as per the country's proclamation, Ethiopia's licensing system by hiring bodies of health professionals was found to be deficient. A system for identifying forged licenses and managing revoked licenses is also lacking in Ethiopian hiring organizations for health professionals.

Despite some knowledge gaps in the category of professionals for whom a license is required during recruitment- the understanding and attitude of Ethiopia's hiring organizations appear to be at a promising level.

By promoting ongoing internal audits among hiring bodies and providing a reporting mechanism to the higher authorities, it is possible to prevent the majority of Ethiopia's public sector health professionals hiring bodies from falling behind the expected licensing practice. Furthermore, if coordinated with internal audits from the hiring bodies for health professionals, rapid supervisory visits from higher authorities aid in the improvement of the licensing practice.

## Data Availability

On reasonable request, the corresponding author will provide the data used and/or analyzed during the study.

## Abbreviations

Adjusted OR: Adjusted Odds Ratio
COC: Certificate of Competence
Crude OR: Crude Odds Ratio
CPD: Continuous Professional Development
WHO: District Health Office
FMHACA: Food, Medicine, and Healthcare Administration and Control Authority
GMC: General Medical Council
GP: General Practitioner
HR: Human Resource
HRH: Human Resource for Health
JHPIEGO: Johns Hopkins Program for International Education in Gynecology and Obstetrics
LE: Licensing Examination
SNNPR: Southern Nations, Nationalities, and Peoples Region
UHC: Universal Health Coverage
USA: United States of America
USMLE: Unites States Medical Licensure Examination
WHO: World Health Organization
ZHD: Zonal Health Department
